# Schrödinger’s *What is Life?*—Complexity, Cognition and the City

**DOI:** 10.3390/e25060872

**Published:** 2023-05-30

**Authors:** Juval Portugali

**Affiliations:** Department of Geography and the Human Environment, The Raymond and Beverly Sackler Faculty of Exact Sciences, School of Geosciences, Tel Aviv University, Tel Aviv 69978, Israel; juval@tauex.tau.ac.il

**Keywords:** delayed entropy, free energy, order out of order and aperiodic crystal, genetic code

## Abstract

This paper draws attention to four central concepts in Schrödinger’s ‘*What is Life?*’ that have not, as yet, received sufficient attention in the domain of complexity: *delayed entropy*, *free energy*, *order out of order* and *aperiodic crystal*. It then demonstrates the important role the four elements play in the dynamics of complex systems by elaborating on their implications for cities as complex systems.

## 1. Introduction

The “official history” of complexity theory started in the mid-1960s with founding theories, such as Prigogine’s dissipative structures, Haken’s synergetics, Mandelbrot’s fractals, Lorenz’s chaos and more. However, as shown in a recent study by Haken [1] on “The emergence of complexity theory…”, the roots of the various theories should be traced back to, e.g., Clausius’ (1822–1888) introduction of the concept of entropy in the 19th century, to Cantor’s (1845–1918) set that preceded Mandelbrot’s (1924–2010) fractal geometry, to the birth of Chaos theory proposed by Henri Poincaré (1854–1912) and, more recently, to Erwin Schrödinger (1887–1961), one of the fathers of quantum mechanics, though not for his contribution to quantum theory (for which he received the Nobel Prize) but for his little book *What is Life* [2]. Schrödinger’s book can be considered a forerunner of complexity theory [3] for directing attention to two properties of life, which are also properties of complexity: *open system* and to the notion of *order out of chaos* (‘order out of disorder’ in his words). Here, I draw attention to four more concepts Schrödinger developed in ‘*What is Life?*’, that, in my view, are central to the complexity of cities but have not, as yet, received sufficient attention—*delayed entropy*, *free energy*, *order out of order* and *aperiodic crystal*.

The discussion below, thus, evolves as follows: It starts with a concise introduction to Schrödinger’s “*What is Life?*” (Section 2) and then explores the four concepts noted above. Section 3 introduces the notion of delayed entropy and explores its implications for complexity and *complexity theories of cities* (CTC). On the one hand, it does so in light of recent studies and findings regarding synergetic cities [4]; on the other hand, Section 3 brings to the fore a novel dimension of complexity not noticed before: that while the interaction with the environment is the source of the system’s survival, it is also the source of its destruction. Section 4 discusses the concept of free energy—its original formulations proposed by Helmholtz and Feynman, its recent re-appearance in the form of Friston’s *free energy principle* (FEP) in the context of neurobiology and its further application to synergetic cities [4]. The innovative part here is a comparison between *the Nich Construction* Theory (NCT), as interpreted by Friston’s FET and Portugali’s [3] notion of cities as hybrid complex systems—a comparison that entails a whole set of research questions and propositions that will have to be explored in future research. Interestingly, while Schrödinger’s ‘Order out of disorder’ became a central motto in the domains of complexity theory and of CTC (as ‘order out of chaos’), his notion of ‘order out of order’ remains unnoticed in both domains. Section 5, which follows, introduces Schrödinger’s ‘order from order’ principle and, for the first time, explores its role in the context of complex systems and of CTC by reference to case studies of cultural evolution and to the space–time diffusion of cities and urbanism. It shows, for the first time, that cities as complex systems provide a par-excellence case of order from order. Finally, Section 6 introduces Schrödinger’s concept of aperiodic crystal that inspired the revelation of DNA and, in light of this, examines several questions about its relevance to the dynamics and evolution of cities and, by implication, of complex systems in general: Do complex systems such as cities have a genetic code? Do they have a different kind of code? Do they have a code at all? The paper concludes by indicating that the latter questions, together with those emerging from previous sections, provide a research agenda for future research directions.

Three methodological clarifications are needed before we proceed: Firstly, the article does not fall into a single predetermined research category (e.g., review vs. research paper). Its aforementioned aim to “draw attention to four concept…”, requires an historical perspective, while elaborations on the centrality of the four concepts to complexity theory and to cities as complex systems require an analytical but also didactic orientation. Secondly, in order to illustrate how the four notions inform complexity theory, the discussion refers heavily to the domain of CTC. This, however, is not just because this is my personal domain of interest but due to the fact that, from the first days of complexity theory, cities were employed as examples for complexity—in Prigogine’s [5] Nobel lecture and since then in connection with all theories of complexity. The prominent role of physicists in the domain of CTC is evidence of this. Thirdly, and related to the above two, is the issue of novelty. While Schrödinger’s book has received considerable attention, his being a forerunner of complexity theory has received much less, while the role of his four principles (that form the core of this paper) in the dynamics of complex systems and of CTC, not at all. Therefore, while this paper reflects on earlier work and does not report on novel findings, it is, nevertheless, still novel in bringing Schrödinger’s four notions to attention as central principles of complex systems and of cities as such; herein lies the main innovation and contribution of this paper.

## 2. Schrödinger’s “*What is Life?*”

*“How would we express the marvelous faculty of a living organism, by which it delays the decay into thermodynamical equilibrium (death)?”*

This is the core question in Nobel laureate Erwin Schrödinger’s [2] little book *What is Life?* Based on a lecture Schrödinger gave a year earlier at Trinity College, Dublin, the book suggests a physicist’s view on the phenomenon of life. From the perspective of physics/thermodynamics, claimed Schrödinger, life is a delayed entropy, in his words: by means of the process of metabolism, a living organism

*“… feeds upon negative entropy, attracting… a stream of negative entropy upon itself, to compensate the entropy increase it produces by living and thus to maintain itself on a stationary and fairly low entropy level.”*

With his notion of *negative entropy* (later termed *negentropy* by Brillouin, [6]) and his suggestion that “Organization is maintained by extracting ‘order’ from the environment”, Schrödinger can be considered as a forerunner of complexity theory [3] for anticipating the notion of ‘order out of chaos’ (“order from disorder” in his words) that has become the motto of complexity theory. There are, however, four additional notions/principles suggested by Schrödinger in ‘*What is Life?*’, which are of relevance to complexity theory (CT) and CTC but have not, as yet, received sufficient attention in the domain of CTC: *delayed entropy*, *free energy*, *order from order* and the *aperiodic crystal* (or *material*). In what follows, I briefly introduce each and explore the relevance and implications for complexity theory and CTC.

## 3. Delayed Entropy

Life, according to Schrödinger, is, thus, a property of a system that manages to *delay* the decay into thermodynamical equilibrium, that is, to *temporarily* delay (not to evade) the process that leads the system toward maximum entropy. How? Through its ability to interact with its environment and, in this interaction, to extract negative entropy (or more accurately, free energy—see below) from the environment. This principle was embraced in complexity theory and generalized to complex systems at large, including cities; in order to survive, a city must interact with its environment.

A nice illustration is Alan Weisman’s [7] thought experiment in his non-fiction book *The World Without Us* that simulates what might happen to the products of human culture, cities included, if we humans suddenly disappear. Weisman’s thought experiment is not related to complexity, but in the parlance of complexity, it can be seen as a simulation of a process in which human society ceases delaying the decay of the various artifacts it created into thermodynamical equilibrium, toward maximum entropy. According to Weisman’s simulation, the pace of deterioration of the different human-made artifacts depends on their structure and material so that cities with their residential neighborhoods will become forests within some 500 years, while artifacts, such as bronze statues, plastic materials and radioactive waste, are likely to be the last evidence of human culture.

### 3.1. Delayed Entropy as a Double-Edged Sword

Weisman’s simulation brings to the fore an interesting novel property regarding the interaction of complex systems with their environment: the common view is that, by means of this interaction, the system extracts negative entropy and, thus, manages to survive, i.e., temporarily delay the process that leads it toward maximum entropy. What Weisman’s example shows is that this interaction is a ‘double-edged sword’; on the one hand, it is the system’s source of survival, yet, simultaneously, it is also the force responsible for the decay of the system into thermodynamical equilibrium toward maximum entropy. Once the system is no longer capable of extracting negative entropy from the environment, the environment, composed as it is of a huge number of organic and inorganic entities, will gradually disintegrate the system and bring it into a state of maximum entropy. The interaction with the environment is, thus, the system’s source of survival and life, yet, at the very same time, also the cause of its disintegration and decay.

The property and view that life and, by extension, complex systems are capable of only temporarily delaying their decay into thermodynamical equilibrium, but not to avoid it, implies the property of *impermanence*, a notion that appears in Eastern and Western philosophies alike from early times, in Buddhist philosophy and in Heraclitus’ becoming vs. Parmenides’ being; nowadays, in the context of complexity theory in Prigogine’s [8] book *From Being to Becoming* and, of course, in the basic property of complex systems, they are in a ‘far from equilibrium condition’, never in rest. What the various complexity theories have added to this perception is that the property of impermanence takes two forms of *pace*: relatively long periods of steady state during which the system changes slowly undergoing occasional minor fluctuations and short periods characterized by strong fluctuations that often lead to a phase transition.

### 3.2. Urban Rhythms

**Figure 1:**
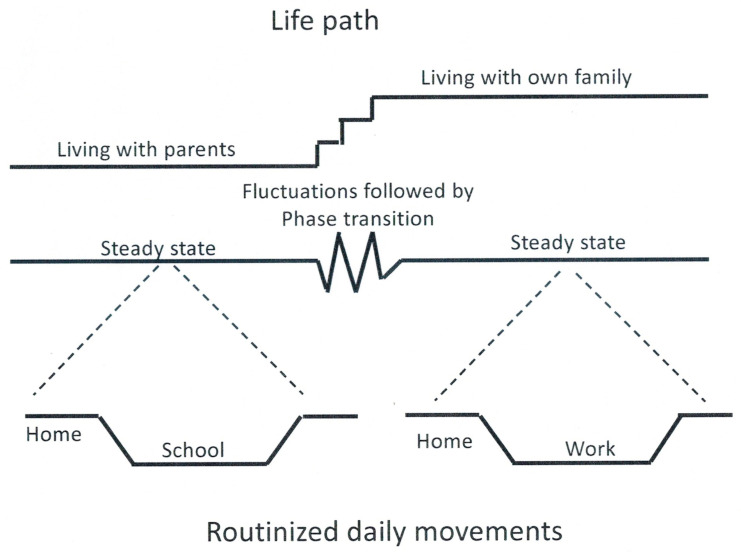
The upper part illustrates the life path of a typical urban agent in terms of movement in space-time (as originally formulated by H in his time geography). The middle part illustrates the life path in terms of a complex system (steady state, fluctuations followed by phase transition and steady state), while the bottom part represents the daily paths as routinized movements during steady state (again in line with H). Source: ([4], Figure 8.2).

The above forms of pace and their manifestation in the dynamics of cities were recently subject to intensive scrutiny by Haken and Portugali ([4], Chapters 8–10 and 15). It was shown that looking at the play between these two forms of pace from the perspective of the *longue durée* of cities reveals an urban rhythm that shows up in the various scales of urban life, ranging from the scale of an individual person to that of neighborhoods, cities, metropoles and more. Figure 1 illustrates this by reference to a single urban agent. It does this by reformulating Hägerstrand’s [9] distinction between a person’s daily and life paths as two facets of a behaving urban agent. While the focus of Figure 1 is on a single urban agent, Haken and Portugali ([4], p. 112) emphasize that such individuals’ behavior transformed into collective behavior becomes the engines of the dynamics of cities: “every morning millions of people wake up more or less at the same time, commute to city centers more or less at the same time, work and then commute back home, once again at more or less at the same time and this is so day after day…” (ibid).

**Figure 2:**
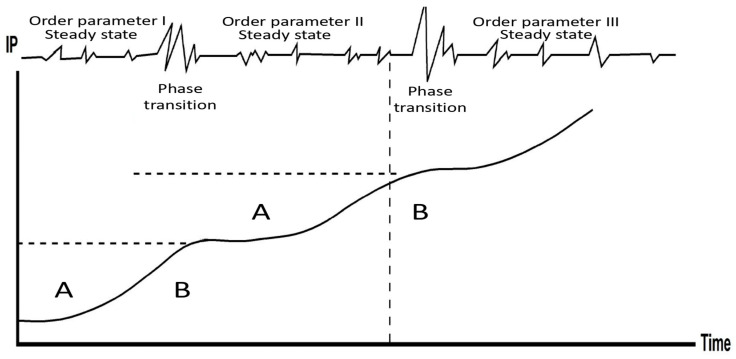
Steady state, phase transition and fluctuations in the *longue durée* of cities as complex systems. In periods A, the city’s order parameter is resilient against, and enslaves, the various fluctuations, while in periods B, the fluctuations intensify and become stronger, thus leading to a phase transition and the emergence of a new order parameter that once again enslaves the system in a new steady state. Ordinate—the city’s information production. For further details, see ([4], Chapter 15).

The resulting pattern at the city scale is illustrated in Figure 2. There are long periods of steady state, during which the spatial behavior of the city’s inhabitants is characterized by a routinized movement and minor scale fluctuations, interrupted by short periods of strong fluctuations that often lead the system to a phase transition and the emergence of a new steady state. Such transformative events might be the result of natural disasters, as was the case of the tragic 2010 earthquake in Port-au-Prince, Hawaii [10,11], but also a consequence of “regular” socio-urban processes such as gentrification ([4], Chapter 10). As we shall see next, the notion of free energy sheds further light on the processes of steady state, while the principle of order from order, on the dynamics of phase transition.

## 4. Free Energy

Central to Schrödinger’s view on life is the notion of negative entropy (negentropy), as we have just seen. Yet, this view of Schrödinger was strongly criticized by fellow physicists—a criticism that he, in fact, accepted. In response, he explained his usage of negative entropy as follows:

*“… if I had been law catering for them alone [e.g., physicist colleagues] I should have let the discussion turn on free energy instead [italics added]. It is the more familiar notion in this context. But this highly technical term seemed linguistically too near to energy for making the average reader alive to the contrast between the two things. He is likely to take free as more or less an epitheton ornans without much relevance, while actually the concept is a rather intricate one, whose relation to Boltzmann’s order-disorder principle is less easy to trace than for entropy and ‘entropy taken with a negative sign’, which by the way is not my invention.”*

### 4.1. The Origin of the Free Energy Concept

As Schrödinger indicates, the concept of free energy to which he refers is “a rather intricate one”. It was originally coined by von Helmholtz (1821–1894) in relation to thermodynamics (the theory of steam engines), while at a later stage, it was elaborated by Feynman [12] in the context of statistical mechanics ([4], Chapter 7). As exemplified and explained by Haken (personal communication),

*“a gas at some temperature contains some inner energy, while gas in a tube with a piston (a steam engine) exerts a force on the piston and can move it doing work. The notion of FE says that only some part of this inner energy can be transformed into work—this part is termed free energy.*

*This law holds quite generally beyond thermodynamics. For example, when we take up food, only part of its inner (chemical) energy can be transformed into the work done by our muscles, similarly to electro-chemical processes that require energy. Applied to cities we would say that the maintenance of a city and its dynamics requires energy in form of high quality energy—the FE, which is only part of the energy actually burned in the urban process; the rest is useless heat.”*

As further shown by [1], in a more general context, free energy can be seen as a ‘terminus technicus’ to characterize a specific mathematical procedure that can be used to calculate energy, e.g., of a ferromagnet; however, in numerous other cases, the quantity to be calculated need not be an energy but can refer to, e.g., numbers of excited neurons or populations in a city, etc. Nevertheless, in all these cases, the formalism is that used by Feynman [12]. A case in point is the income distribution in a city that can be interpreted as a Gaussian probability distribution, with q as the income variable and f as a free parameter. Free energy then allows one to calculate f with increasing precision.

In the last few decades, the free energy formalism defined by Feynman [12] “re-appeared” in the domain of neuroscience in Friston’s notion of the *free energy principle* (FEP). More recently, and inspired by FEP, it was related to urban dynamics in the context of synergetic cities ([4], Chapter 8), in particular regarding the play between steady state and phase transition.

### 4.2. Friston’s Free Energy Principle (FEP)

Employing Feynman’s [12] definition of free energy, Friston’s [13] FEP suggests that

*“… that any selforganizing system that is at equilibrium with its environment must minimize its free energy… The principle is essentially a mathematical formulation of how adaptive systems resist a natural tendency to disorder… in the face of a constantly changing environment”.*

Referring to cognitive processes, the principle further suggests (ibid) that

*“… biological agents must avoid surprises… [ and, that] free energy is an upper bound on surprise, which means that if agents minimize free energy, they implicitly minimize surprise”.*

**Figure 3:**
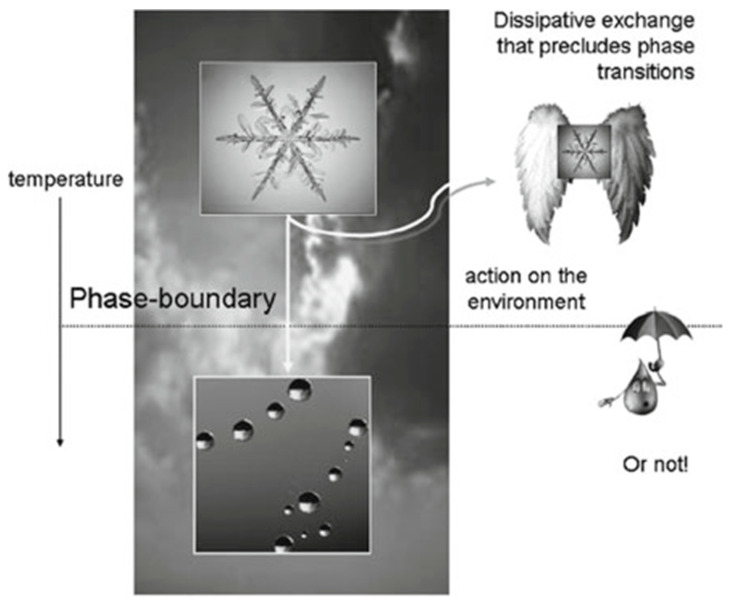
“… By occupying a particular environmental niche, biological systems can restrict themselves to a domain of parameter space that is far from phase-boundaries. The phase-boundary depicted here is a temperature phase-boundary that would cause the snowflake to melt (i.e., induce a phase-transition)…” ([12], Figure 1). For further information, see ([4], Chapter 7 and Figure 7.1).

To convey this idea, Friston refers to an imaginary creature—a snowflake with wings (Figure 3). In order to survive (i.e., not to melt), such a complex adaptive system must restrict itself “to a domain of parameter space that is far from phase-boundaries… that would cause the snowflake to melt…”, that is, to undergo phase transition. Similar to the imaginary snowflake, animals such as birds routinely move thousands of kilometers each year in order to remain in appropriate ecological conditions and, thus, to avoid surprises.

Friston is, of course, fully aware of Schrödinger’s *What is Life?* In a paper entitled “Answering Schrödinger’s question: A free energy formulation” [14], he and his group suggest that FEP is an answer to Schrödinger’s question. They do not refer, however, to Schrödinger’s usage of free energy but, rather, to his observation that living systems appear “to resist the 2nd law of thermodynamics by persisting as bounded, self-organizing systems over time” (ibid, 1).

### 4.3. The FEP and Its Relations to Cities

**Figure 4:**
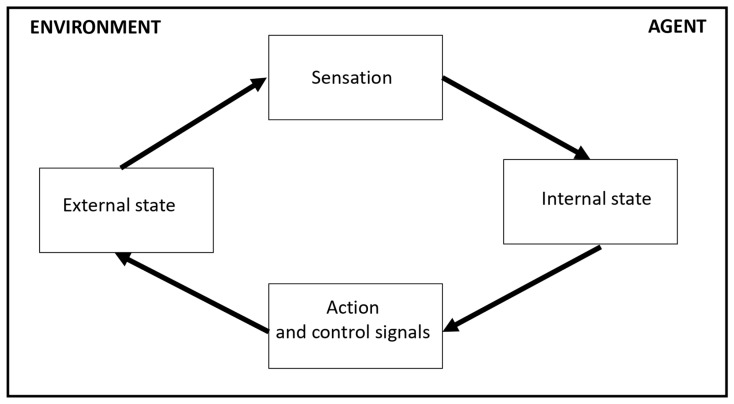
According to Friston’s FEP, CASs maintain their steady state by means of an ongoing perception–action play between their *external states* and their *internal states*. A simplified version of Box 1 in [13].

As can be seen, Friston’s FEP refers, and adds insight, to the dynamics of steady states, suggesting that CASs (complex adaptive systems) have an innate tendency to avoid phase transition and perpetuate their steady state—physically, by restricting themselves to ecological “niches” that enable them to maintain their steady state, and mentally, to ‘cognitive environments’ that enable them to avoid surprise (that is, a cognitive phase transition). The latter is implemented through an ongoing *perception*–*action* play between their *external states*, the data flow from the environment that generates sensory samples, and their *internal states*, the neural activity responsible to the recognition regarding the cause of a particular sensation (Figure 4).

As is well recorded, the *longue durée* of complex self-organizing systems is characterized by long periods of steady state interrupted by short periods of strong fluctuations that often entail a phase transition. This has led to the impression “that the free energy principle precludes novelty and complexity, because it assumes that biological systems… try to minimize… surprise [in order] to maintain their homeostasis” [15]. To correct this impression and account for the overall behavior of complex systems, Friston and his team (ibid) made a link to the so-called *exploration*–*exploitation dilemma* [16], showing that in exploitation, Bayesian surprise should be reduced, while in exploration, phase transition is necessary. I am indebted to an anonymous reviewer of this article for drawing my attention to this point. This, thus, “dissolves any dialectic between minimizing surprise and exploration or novelty seeking” [15].

Despite the ‘E’ in Friston’s FEP, this principle does not refer to the role of energy in such processes, and it is not involved in the “energetic” details. This is apparently due to the very complicated pathway in life processes, from the elementary energy processes to the final result, in the form of muscle contraction, sensations and the like. As a model that employs mathematical tools from physics, FEP, thus, deals with relations between macroscopic features, e.g., perception and action in the case of cognition, or in the context of cities, the relations between the number of citizens and income. For details, see ([4], Chapter 8).

Friston’s FEP refers mainly to biological complex systems. However, from his FEP perspective, cities can be likened to ecological niches specifically constructed by humans in order to maintain their innate tendency for steady states, physically by constructing and reproducing the material structure of cities (buildings, roads, etc.), while cognitively by means of the routinized and reproductive behavior of urban agents that minimizes surprise, thus avoiding cognitive phase transition (as described in Figure 1, bottom).

It is interesting to note that, in recent years, the process of niche construction has been suggested as an integral component in standard evolutionary processes such as natural selection. This view, termed *niche construction theory* (NCT) [17,18], entailed much debate in biology, including a study by Friston’s group suggesting their variational FE approach as a theoretical framework for niche construction [19]. Can NCT be extended to the domain of cities? While a full-scale discussion about NCT and urban theory will have to await another study, it can be emphasized here that there is a fundamental difference between biological niches and cities as “niches”.

Cities are hybrid complex systems composed of artifacts (objects constructed by humans), which are simple systems, and humans as complex systems [3,20,21]. Now, NCT too refers to objects that are constructed by animals (e.g., a bird’s nest), though these are not artifacts (ibid) but rather parts of nature. There is, thus, a fundamental difference between a bird’s nest and a human building (e.g., a house) and, in a similar way, unlike animals whose niches are integral parts of nature, cities as niches are at least partly artificial and are not part of nature. One of the key differences follows the fact that natural niches are subject to the slow process of Darwinian evolution, while artifacts are the stuff of cultural evolution and are, thus, subject to the fast process of cultural evolution. The notion of order out of order that is discussed next sheds light on the specific dynamics of cities.

On the face of it, the above view that the artificial components of cities as hybrid complex systems are simple systems contradicts previous views about artifacts, specifically Simon’s [22] view in his work *The Sciences of the Artificial*, that artifacts designed and made by humans are complex systems. Yet, this is not the case—there is no contradiction here: complexity theory started by reference to phenomena of material systems, such as the *Bénard* cells or the LASER. This physicalist approach was successfully applied to organic and social systems that were also shown to be complex systems. However, at a later stage, the studies of Gell-Mann [23,24] and Holland [25] demonstrated that due to the property of adaptation that typifies life, living complex systems differ from material ones in being at once complex and *adaptive* systems (hence, the notion of CASs). In a similar way, it has been demonstrated that while cities are complex systems and CASs, they still differ from the latter two in also being hybrid complex systems, as defined above. It must be emphasized that cities are but one example of a much larger class of hybrid complex systems, an issue that extends beyond the context of the present study.

## 5. Order from Order

As noted above, the property of *impermanence* implied by Schrödinger’s view of life follows human thinking from early times. However, while there is no room here to delve into this topic, it is interesting to note that the impermanence of cities as complex systems, as above, entails a dilemma: how would we explain, for instance, firstly, the existence of the complex system ‘city’ for more than 5000 years now, and secondly, the differences between the various cities that existed in these thousands of years? The answers come from Schrödinger’s ‘order from order’ principle.

Schrödinger’s ‘order out of disorder’ principle anticipated the ‘order out of chaos’ motto of complexity theories. Yet, Schrödinger considered the latter principle as secondary, suggesting that life springs from another principle that he termed *order from order*:

*The orderliness encountered in the unfolding of life springs from a different source. It appears that there are two different ‘mechanisms’ by which orderly events can be produced: the ‘statistical mechanism’ which produces order from disorder and the new one, producing order from order.*

While the first is in line with the laws of physics, continues Schrödinger, it is not sufficient to account for life:

*But we cannot expect that the ‘laws of physics’ derived from it suffice straightaway to explain the behaviour of living matter, whose most striking features are visibly based to a large extent on the ‘order-from- order’ principle.*

The ‘order from order’ principle follows the observation that “… the gene generated order from order in a species, that is, the progeny inherited the traits of the parent”. Cities as complex systems provide a par-excellence case of order from order, namely, that the “progeny city” inherited the traits of the “parent city”.

### 5.1. The “Progeny City” Inherits the Traits of the “Parent City”

Cities, as is well recorded, have existed for more than 5500 years now. During this time, other social formations, such as empires, kingdoms, clans, tribes…, emerged, declined and disappeared, while cities not only still exist but have become the main form of settlement and life. During these last 5500 years, cities have changed from generation to generation, from culture to culture and from place to place, when the principle and engine of their change has not been order out of chaos (the title of [26]) but rather Schrödinger’s order from order principle. The latter principle is commonly discussed in connection with the space–time diffusion of species but, as we will see next, it is also associated with the space–time diffusion of cities and urbanism.

### 5.2. The Space–Time Diffusion of Cities and Urbanism

**Figure 5:**
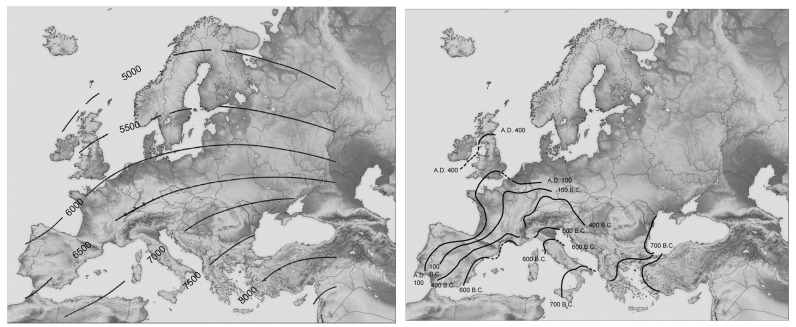
***Left***: The spatial diffusion of agriculture from its core of origin in the Middle East westward. The isolines represent time BP. ***Right***: The spatial diffusion of urbanism westward (mainly in the Roman period). See further discussion in [4], Chapter 11.

Cities, as complex hybrid systems, are composed of artifacts (simple systems) and humans (complex systems). Now, artifacts of all kinds, including tools, machines, cars, buildings, roads and whole cities, are subject to *cultural evolution*—a process that attracted intensive research [27]. Of specific relevance here is Cavalli-Sforza and Feldman’s [28] study *Cultural Transmission and Evolution*: *A Quantitative Approach*. Commencing from a neo-Darwinian point of view, they propose that just as biological evolution is governed by *genetic transmission*, so cultural evolution is governed by *cultural transmission*. However, Cavalli-Sforza and Feldman (ibid) further suggest that in order to apply the neo-Darwinian perspective to cultural evolution, one has to take into consideration one difference between the two transmission processes: Unlike biological transmission, which occurs between the parent and child of successive generations only, in the cultural domain, transmission takes place between the parent and child of successive generations, between neighbors and between neighbors of two successive generations. These they termed, respectively, *vertical*, *horizontal* and *oblique* transmissions. Similar to “copying mistakes” (i.e., mutations) in genetic transmission, in culture, a new cultural trait is born out of *learning mistakes*. Finally, as a genetic mutation becomes subject to environmental selection, so a newborn cultural mutation becomes subject to selection by the “cultural environment” that determines its fate to extinction or *spatial diffusion*. As an example, Cavalli-Sforza and Feldman reconstructed the spatial diffusion of agriculture (Figure 5, *left*). In Figure 5, *right*, the spatial diffusion of urbanism was added.

However, cultural evolution differs from biological evolution in three additional fundamental (and interrelated?) respects: the *pace* of the two processes, as just discussed, the role of memory and imagination in the process and the question of *code*, namely, do artifacts have a cultural code similar to the genetic code of organic entities? The first two issues are discussed next, while the third is discussed in Section 5.

### 5.3. The Pace of Cultural Evolution

Compared to biological evolution, cultural evolution (that is, the time–space diffusion of cultural traits) is an extremely fast process. While fast, there are, however, considerable time differences between the various processes of cultural evolution. Thus, the spatial diffusion of agriculture and urbanism (Figure 5) are on the time-scale of thousands of years, whereas that of the new ICT (information communication technology) gadgets are on the time-scale of months. The speed of adoption depends, on the one hand, on the nature of the cultural trait (e.g., urbanism vs. smart phone or coronavirus), while on the other, on the properties and spatial distribution of the potential adaptors. For example, in the case of agriculture and urbanism, the process implied that more and more socio-spatial–cultural communities had to undergo their specific cultural revolution—a phase transition in the parlance of complexity: from hunting–gathering to agriculture in the case of the Neolithic/Agricultural revolution, and from nomadic or sedentary agriculture to cities in the case of urban revolution. The outcome is the space–time diffusion of urban society, for instance, evolving as a sequence of smaller-scale phase transitions (i.e., revolutions), when each such community underwent its own specific urban revolution. For archaeological evidence of such a process, see [21].

### 5.4. Cities and Memory

However, what about the first city? What principle gave rise to its emergence some 5500 years ago? It is commonly agreed that cities and urban society came into being via an event termed by Gordon V. Childe [29] as *urban revolution*. It is also commonly agreed that this event followed the collapse of the Neolithic agricultural society that preceded it. This Neolithic culture dominated for some 5000 years since the so-called Neolithic revolution around 10,000 years B.P. and then collapsed and was replaced by the emerging urban society. On the face of it, the event of the *urban revolution* must be considered a case of an emerging new order out of disorder, since the collapse of the Neolithic era with its agricultural society was likely to be associated with a chaotic period. Indeed, the various theories about this event agree that the Neolithic period ended up with a major crisis and chaotic reality, yet they disagree and differ in their suggestions regarding the factors that brought about the collapse. According to Childe’s (ibid) Marxist view (or rather speculation), neolithic society collapsed as a consequence of the urban revolution, itself a logical consequence of the neolithic mode of production: food production entailed surplus and specialization, which gave rise to a stratified society and the emergence of urbanism. More recent studies suggest that the cause might have been a large-scale plague [30]; as just noted, the specific causes are still largely debated [31,32].

The evidence about the emergence of urban society some 5500 years ago is often associated with the implicit or explicit view that cities and urbanism were a new invention—an outcome of the phase transition between the neolith and urbanism. However, as recently shown [32], archaeological excavations in sites such as Chatal Huyuk indicate that the city as a form of settlement and way of life was known some 3000 years before the urban revolution, with the implication that the cities of the urban revolution were not a new invention but rather a reconfiguration of known, rarely used, socio-spatial forms of settlement that were ‘stored” in society’s collective memory. When the overall situation changed, these, so far marginal, forms of settlement were extracted and used as the new dominant form of living. In short, the urban revolution was the realization of a potential or knowledge that was stored in memory. The same was the case with the agricultural–neolithic revolution, the essence of which was the domestication of plants and animals. Archaeological findings indicate that the knowledge about, and the technology of, domestication was known but not employed for a long time before the agricultural revolution, some 10,000/12,000 years B.P.

In light of the above, the urban revolution of 5500 years ago can also be considered a case of order from order. Therefore, do we have to give up the order out of chaos principle? No! The two principles co-exist. The *longue durée* of human settlement, for instance, is characterized by long periods of steady state interrupted by short periods of chaos, phase transition and the emergence of a new order and a new steady state and so on. Thus, assume that in the upper part of Figure 2, the first steady state, dominated by order parameter I, refers to a society of hunters and gatherers; order parameter II, which dominates the second steady state, refers to an agricultural/neolithic society, while order parameter III dominates the steady state of an urban society. Thus, on a wider time scale, the relations between the three steady states follow the order from order principle, while on a shorter time scale, when focusing on each single steady state, the principle of order from disorder (chaos) takes the lead.

## 6. The Aperiodic Crystal

“… let me anticipate”, writes Schrödinger, “what will be explained in much more detail later, namely, that the most essential part of a living cell-the chromosome fibre may suitably be called an aperiodic crystal. … which, in my opinion, is the material carrier of life.” By the notion of aperiodic crystal or solid, Schrödinger refers to an ordered and organized, but non-periodic, structure of chromosomes that contain “… in some kind of code-script the entire pattern of the individual’s future development and of its functioning in the mature state. Every complete set of chromosomes contains the full code.” He then concludes:

*We might quite properly call that an aperiodic crystal or solid and express our hypothesis by saying: We believe a gene -or perhaps the whole chromosome fibre -to be an aperiodic solid.*

Less than 10 years later, in 1953, inspired by Schrödinger’s brilliant intuition, Cambridge University scientists James D. Watson and Francis H.C. Crick announced that they had determined the double-helix structure of DNA, the molecule containing human genes. “Watson and I” wrote Crick to Schrödinger on the occasion of his 66th birthday, (12 August 1953),

*“… were once discussing how we came to enter the field of molecular biology, and we discovered that we had both been influenced by your little book ‘What is Life?’… We thought you might be interested—you will see that it looks as though your term ‘aperiodic crystal’ is going to be a very apt one.”* (Quoted in [33], Chapter 4).

Why did Schrödinger term the code-script of life (later termed DNA) aperiodic crystal or solid? Apparently, it was to emphasize the way it differs from a regular (periodic) crystal, whose growth is characterized by periodicity in which a given motif (a unit cell) repeats itself again and again in 3D space. An aperiodic crystal or solid, per contra, lacks this strict periodicity. Compared with the aperiodic crystal, writes Schrödinger, periodic crystals

*are rather plain and dull. The difference in structure is of the same kind as that between an ordinary wallpaper in which the same pattern is repeated again and again in regular periodicity and a masterpiece of embroidery, say a Raphael tapestry, which shows no dull repetition, but an elaborate, coherent, meaningful design traced by the great master.*

As noted by Varn and Crutchfield ([34], p. 3),

*… exact repetition of a motif, in other words a crystal, is information poor—too poor to carry heredity. Without some unpredictability, or novelty, nothing new is learned and communicated. It is remarkable that Schrödinger made this prediction before a quantitative understanding of information was articulated.*

Schrodinger’s aperiodic crystal, thus, emphasizes unpredictability, impermanence and novelty. On the other hand, however, as we have seen above, Friston’s FEP emphasizes the exact opposite—the tendency of complex living systems to maintain a steady state. How can these two perceptions and properties be reconciled? Can they be reconciled? The answer is positive if we look at the *longue durée* of settlement and cities, as above, that are characterized by long periods of steady state (little change) and short events of phase transition (drastic major change).

The genetic code is a property of living systems, and living systems are complex systems. Yet, not all complex systems are living systems and, as such, need not have a genetic code. What about cities as complex systems?

### 6.1. Do Cities Have a Genetic Code?

One of the surprises that followed the finding of the genetic code, the structure of DNA and the possibility to measure genetic distances, concerns the degree of similarity within and between species: 99.9% of your DNA is identical to that of any other human being, while 98.8% of our DNA is identical to that of chimpanzees. We humans are not so different from each other and from other animals as we tend to think and experience. Can this be applied to cities? Is there a genetic code of all cities? Can we say that the similarity between the cities of 5000 years ago is 99%, 98% or even 70% identical to the cities of today? According to Hillier [35] the answer is positive—finding the genetic code of cities might be “simpler than we think” declares the title of his paper.

In this paper, Hillier uses his space syntax formalism as a means to explore the possibility that there exists a universal “genetic code” for cities that will provide the foundation for “a theory of a universal city underlying cities in general…” Hillier’s space syntax, as developed by him in the last few decades, refers specifically to the functional and spatial structures of cities and the relation between them [36]. Commencing from his space syntax approach, Hillier [35] proposes “a new universal definition of a city as a network of linked centres at all scales set into a background network of residential space”. More specifically, he describes cities in terms of two networks: a foreground network of relatively few long lines that connect activity centers in the city and a background network of many short lines, characterizing the residential space of the city. He further shows that, in line with space syntax, the above “universal pattern comes about in two interlinked but conceptually separable phases: a spatial process through which simple spatial laws govern the emergence of characteristically urban patterns of space from the aggregations of buildings; and a functional process through which equally simple spatio-functional laws govern the way in which aggregates of buildings become living cities.” This dual process, he suggests, “can lead us in the direction of a ‘genetic’ code for cities”.

Hillier then notes that space syntax studies brought to light two phenomena that, together, according to him, make up the genetic code of cities: spatial emergence and spatial agency. Spatial emergence is an elaboration of the structuralist view of the city as a representation of socio-economic processes. However, in place of the structuralist view that space (e.g., the city) is a representation of socio-economic processes, he suggests that socio-economic processes give rise to specific acts of building that, in turn, shape the urban space. By spatial agency, Hillier refers to the functional ‘from space to society’ processes by which the structure of the emerging space affects, for instance, movement flows that, in turn, affect land use (as proved time and again by space syntax) and transform the city from a collection of independent structures to a living city.

“It is these two linked processes of spatial emergence and spatial agency that set in train the self-organising processes through which cities acquire their more or less universal spatial form.” What these self-organizing processes are is not specified in the paper, which, as Hillier emphasizes, is not as yet a fully fledged theory. However, in concluding his paper, Hillier expresses optimism that it “seems reasonable to advance the suggestion than that by expressing the complex processes of self-organisation through which cities come into existence as both spatial and functional systems, in terms of two simple, mathematically expressible laws, we are likely to be close to formulating the principles of a genetic code for cities. It is of course far from complete, and above all in need of a general mathematical treatment”.

### 6.2. Do Cities Have a Memetic Code?

Hillier uses the notion of the genetic code metaphorically, of course, as cities are not a consequence of biological information caried by polymers composed of polynucleotide chains that coil around each other to form the double helix. Can such a metaphor be justified? In light of the above discussion, it can be said that Hillier refers, in fact, to what we have described above as cultural code. If so, it is relevant to note here, firstly, that the cultural analogy to genes and genetic code was already suggested and developed by Dawkins [37] and others in what he termed memes and memetic code, based on an analogy between the genes as biological replicators and memes as cultural replicators. Secondly, the notions of memes, memetic codes and cultural codes have already been applied to the study of cities as complex systems, specifically in connection with urban simulation models and decision making in the context of urban planning [20].

There is no room here to further elaborate on the above applications; however, what is important to emphasize in the present context is the lesson from the above applications: namely, that the analogy between genes and memes is only partial. As already noted above, memes are not genes. They are not innate nor are they associated with a specific material entity such as DNA molecules. Rather, they are products of a complex interaction between internal brain entities—memes and their external manifestations in the form of bodily activities. As such, unlike genes, they quite often represent natural and artificial objects, categories and other entities and external representations that exist in the world. However, most importantly, the relations between genes and their external representations (the parallels of natural phenotypes) are asymmetric. In biological evolution, genotypes give rise to externally represented phenotypic effects, but the latter cannot be transformed back into genotypes. Memes and their externally represented (“phenotypic”) effects, per contra, co-exist in symmetric relations. An idea about a circle can generate a circle in the world, while an object in the world, say a shelter, can generate the memes ‘home’ or ‘house’. This is the case with respect to a house as a single artifact, and this is the case with respect to a group of houses that forms the meme ‘neighborhood’ and, on a larger scale, the meme ‘city’.

### 6.3. Do Cities Have a Code?

Why the question mark? Why doubt the possibility that, similarly to organisms, cities too have a code, if not genetic then memetic? This is the case, since the view of the city as a kind of organism accompanies the study of cities from the first day urbanists began to map and describe cities [38,39]. Part of the answer was already given above: cities are hybrid complex systems composed of human agents, each of which is a complex system, and artifacts, which are simple systems. Artifacts, as noted, are the staff of cultural evolution, driven as they are by the process of cultural transmission, which is much faster than that of genetic transmission. Now, do artifacts, cities included, have a code?

Both notions—genetic and memetic codes—imply that at the core of each member of a category (homo sapience, chimpanzee, other animals), there is a common code that is shared by all members of that category. In the case of cities, it implies that all cities from the last 5500 years must share a common set of properties that form their genetic or memetic code. Wittgenstein’s [40] notion of *family resemblance* and subsequent cognitive theories of categorization challenge and question this view with respect to categories in general, while in several past studies [20,41], it has been shown that these challenges apply to cities too. Namely, while cities have existed for more than 5500 years, due to their artificial component, they do not have (or need not have) a genetic or memetic code; their only common denominator is the name “City”.

### 6.4. Wittgenstein’s Family Resemblance and Its Implications

In paragraph 66 of his *Philosophical Investigations*, Wittgenstein [40] uses the example of the category “game” in order to challenge the dominant view that all members of a given category must share a common set of properties that form the necessary and sufficient condition for a member to be included in that category, which, in the parlance of the present discussion, is that all members of a given category must share a common memetic/cultural code in order to be included in that category. According to Wittgenstein (ibid), many categories come into being as a consequence of a process by which their members form what he termed ‘family resemblance networks’, “a complicated network of similarities overlapping and criss-crossing: sometimes overall similarities, sometimes similarities of detail”. The result is that it is possible that two members of that category will share no common properties—no memetic or cultural code. As shown [20,41], cities form a category, not by virtue of a common memetic or cultural code but rather by a family resemblance network that has extended in space and time since the emergence of cities and urban society some 5500 years ago (or even earlier) until the present day.

Wittgenstein’s “family resemblance” was adopted by cognitive scientists as a starting point for a discussion about the processes of categorization and concept formation, specifically in connection with the *embodied cognition* approach—an approach that challenged the domination of the classical cognitivism approach by variously rejecting the brain as a computer by emphasizing the significance of an agent’s physical body in cognitive abilities. Thus, Rosch et al. [42] added that, while many categories indeed form family resemblance nets, some instances of such categories are more typical or *prototypical* so that an orange, for instance, is “more” of a fruit than a melon. Applied to cities [20,41], while cities form a family resemblance network, still, in each space-time moment, it is possible to identify cities which are specifically (proto)typical = they are ideal type.

Rosch et al.’s views on categorization were taken to their logical conclusion through the embodied cognition approach [43]. According to the latter, the very existence of family resemblance categories challenges the *information processing approach* that has dominated cognitive science since its birth in the mid-1950s, with the view that cognition is essentially an algorithm executed on the hardware of the brain, principally independent of the body and its environment. In contrast to this view, proponents of embodied cognition have demonstrated that cognition is embodied. They have done so from a variety of perspectives ranging from psychology and linguistics, to neurology with direct association to complexity and self-organization. (For general reviews see [43,44,45]). Of the latter perspectives, Lakoff and Johnson’s [43] “experiential realism” approach is specifically relevant here. One of its claims is that many family resemblance categories have a core–periphery structure, with the prototypical best exemplars of the category at the core, from which they are related to other peripheral instances of the category by means of similarities, metaphors and family resemblances. From this perspective of embodied cognition, the city is perceived as

*a huge family resemblance network of connected similarities. In this evolving and diffusing network, one can identity core and periphery: cities, images, and urban phenomena, which in a certain space-time moment capture the central area of the net as the most typical or prototypical exemplars as well as cities that form the periphery. They are all related to each other by means of imagination, that is, similarities, metaphors and family resemblances. Cities looked upon from this perspective have been termed “The cities of experiential realism”* ([41], Chapter 1).

## 7. Concluding Notes

The aim of this paper was, firstly, to draw attention to four concepts developed by Schrödinger in his ‘*What is Life?*’ that have not, as yet, received sufficient attention in the domain of complexity: *delayed entropy*, *free energy*, *order out of order* and *aperiodic crystal*. Secondly, the aim was to demonstrate the important role the four elements play in the dynamics of complex systems by elaborating on their implications for cities as complex systems. Accordingly, the discussion in this paper was essentially introductory; namely, each section shortly introduced one of the four concepts and indicated its potential. The next step would be to elaborate on each of the above four notions and materialize their potential in full, that is, firstly, the potential delayed entropy to shed light on the constructive and destructive dimensions of a system’s interaction with its environment, as well as on its association with the notions of impermanence and time–geography and, secondly, the potential of free energy to shed light on the processes of steady state and phase transition in complex systems and in cities as such, and the potential to link the niche construction theory to urban dynamics. Thirdly, the principle of order from order should be considered and its relations to processes of space–time diffusion and the role of memory in them. Finally, the concept of aperiodic crystal entails a whole set of questions regarding whether cities have a genetic code, or a memetic code, or similarly to Wittgenstein’s family resemblance categories, they do not have a code at all. Fascinating as they are, materializing the above potential and answering the above questions will have to await further research.

## Data Availability

Not applicable.
